# Cardiovascular Sequelae of Bronchopulmonary Dysplasia in Preterm Neonates Born before 32 Weeks of Gestational Age: Impact of Associated Pulmonary and Systemic Hypertension

**DOI:** 10.3390/jcdd11080233

**Published:** 2024-07-26

**Authors:** Pramod Pharande, Arvind Sehgal, Samuel Menahem

**Affiliations:** 1 Monash Newborn, Monash Children’s Hospital, 246 Clayton Road, Clayton, Melbourne, VIC 3168, Australia; arvind.sehgal@monashhealth.org; 2 Department of Pediatrics, Monash University, Melbourne, VIC 3800, Australia; samuel.menahem@monash.edu; 3 Paediatric and Foetal Cardiac Units, Monash Medical Centre, Melbourne, VIC 3168, Australia; 4 Murdoch Children’s Research Institute, University of Melbourne, Parkville, VIC 3052, Australia

**Keywords:** preterm infants, bronchopulmonary dysplasia, pulmonary hypertension, systemic hypertension, arterial stiffness, echocardiography, ACE inhibitor

## Abstract

Bronchopulmonary dysplasia (BPD) remains the most common respiratory disorder of prematurity for infants born before 32 weeks of gestational age (GA). Early and prolonged exposure to chronic hypoxia and inflammation induces pulmonary hypertension (PH) with the characteristic features of a reduced number and increased muscularisation of the pulmonary arteries resulting in an increase in the pulmonary vascular resistance (PVR) and a fall in their compliance. BPD and BPD-associated pulmonary hypertension (BPD-PH) together with systemic hypertension (sHTN) are chronic cardiopulmonary disorders which result in an increased mortality and long-term problems for these infants. Previous studies have predominantly focused on the pulmonary circulation (right ventricle and its function) and developing management strategies accordingly for BPD-PH. However, recent work has drawn attention to the importance of the left-sided cardiac function and its impact on BPD in a subset of infants arising from a unique pathophysiology termed postcapillary PH. BPD infants may have a mechanistic link arising from chronic inflammation, cytokines, oxidative stress, catecholamines, and renin–angiotensin system activation along with systemic arterial stiffness, all of which contribute to the development of BPD-sHTN. The focus for the treatment of BPD-PH has been improvement of the right heart function through pulmonary vasodilators. BPD-sHTN and a subset of postcapillary PH may benefit from afterload reducing agents such as angiotensin converting enzyme inhibitors. Preterm infants with BPD-PH are at risk of later cardiac and respiratory morbidities as young adults. This paper reviews the current knowledge of the pathophysiology, diagnosis, and treatment of BPD-PH and BPD-sHTN. Current knowledge gaps and emerging new therapies will also be discussed.

## 1. Introduction

Preterm infants have the challenging task of gas exchange utilising a respiratory system that is still developing. Extreme preterm infants (born at <28 weeks of gestational age (GA)) have lungs still at the canalicular stage, not achieving the alveolar stage until 36 weeks [1]. To sustain life, such infants are dependent on prolonged ventilation and a higher ambient oxygen. These interventions tend to lead to lung injury arising from ongoing inflammation causing disruption to lung angiogenesis and vasculogenesis [2]. Many such preterm infants develop deranged lung function resulting in a significant morbidity and mortality through the development of bronchopulmonary dysplasia (BPD) and resultant pulmonary hypertension (PH (BPD-PH)) [3]. Early and prolonged postnatal exposure to a chronic hypoxic environment induces pulmonary hypertension with characteristic features of a reduced number and increased muscularization of small pulmonary arteries, causing a rise in the pulmonary vascular resistance (PVR) and reduced vascular compliance [4,5]. Chronic hypoxia and the resultant inflammation lead to another less well studied complication of BPD, namely systemic hypertension (sHTN (BPD-sHTN)) [6]. The aim of the paper is to review current knowledge of the epidemiology, pathogenesis, diagnosis, and current therapeutic options of BPD focusing on BPD-PH and BPD-sHTN. Current knowledge gaps, emerging therapies, and future research directions for these conditions will also be discussed.

## 2. The Burden of BPD

BPD remains the most common respiratory sequel of prematurity for infants born at <32 weeks. BPD is currently defined by the Australian New Zealand Neonatal Network (ANZNN) as a continued need for any form of respiratory support (supplemental oxygen and/or assisted ventilation) at 36 weeks of post-menstrual age (PMA) [7]. Jensen and colleagues have graded the severity of BPD based on the degree of respiratory support regardless of oxygen requirement as Grade 1: nasal cannula ≤ 2 L/min; Grade 2: nasal cannula > 2 L/min or non-invasive positive airway pressure; and Grade 3: invasive mechanical ventilation [8]. A recent ANZNN report noted that the incidence of BPD continues to be high (approximately 52%) in infants at ≤27 weeks GA [7]. Although survival has increased for extreme premature infants (22–28 weeks GA), approximately 42% develop BPD each year in the United States, creating an immense burden on healthcare resources [9,10].

## 3. Pulmonary Hypertension Secondary to BPD (BPD-PH):

Pulmonary hypertension is a known complication of BPD. PH occurs in 17–24% of BPD patients [11,12,13]. A recent meta-analysis confirmed the association of PH relative to the severity of the lung disease with a prevalence of PH in 6%, 12%, and 39% of infants with mild, moderate, and severe BPD, respectively [14,15]. The incidence of PH increases as GA decreases, rising to 59% in infants born at or prior to 25 weeks GA [16]. BPD-PH is associated with a high mortality between 14% and 38% [12,15]. Abman and colleagues from the North American Pediatric Pulmonary Hypertension Network (PPHNet) registry reported that BPD-PH was a major health issue in 22% of all PH patients [17]. Although elevated pulmonary arterial pressures have been shown to persist until hospital discharge [11], there is limited information on the long-term outcomes for survivors of BPD-PH. This observation has prompted an urgent call for research to better understand the pulmonary and neurodevelopmental outcomes of this disorder [18].

### 3.1. Pathogenesis of BPD-Associated Pulmonary Hypertension

The pathogenesis of BPD-PH is multifactorial and reflects the impact of antenatal and postnatal injury on lung vascular growth. The maternal and prenatal events impacting foetal lung vascular development leading to PH may be mediated through the placenta. Placental histopathology and biomarkers have been investigated in BPD development. The presence of maternal vascular underperfusion of the placenta is associated with an increased risk of the development of BPD-PH [19]. Abnormal placental vascular structures in the form of vessel maldevelopment and decreased villous vascularity in preterm neonates are strongly associated with the development of BPD-associated PH [20,21]. In addition, the levels of pro-angiogenic growth factors are decreased in the cord blood of preterm infants who have placental vascular malperfusion and who later develop BPD-PH [22].

The disruption of foetal lung development, perinatal infection/inflammation, mechanical ventilation, and hyperoxia may all lead to pulmonary inflammation that deleteriously impacts on both the pulmonary airways and alveoli, as well as their vasculature [23,24,25,26,27]. The maturation of the pulmonary vasculature and the lung parenchyma occurs concurrently. The proximal pulmonary arteries develop via vasculogenesis, whereas the pre-acinar arteries develop via angiogenesis [23]. Dysregulation of the processes underlying blood vessel growth is thought to be an early contributing factor to the development of PH [28]. Vascular endothelial growth factor (VEGF) is an important regulator of angiogenesis and vasculogenesis, mediating the normal development of the pulmonary vasculature [23,29]. Nitric oxide (NO) has also been implicated in normal vascularisation, playing important roles in stimulating endothelial proliferation through the vascular endothelial growth factor–nitric oxide (VEGF-NO) pathway [29,30]. Disruption of the VEGF-NO pathway leads to the impairment of pulmonary microvascular and alveolar formation, contributing to BPD-PH [29]. Dysregulation in lung growth results in a reduced surface area contributing to a ventilation perfusion (V/Q) mismatch, which exacerbates hypoxaemia and hypercapnia and contributes to an increase in the PVR via hypoxic vasoconstriction [31].

### 3.2. Risk Factors

A meta-analysis and systematic review identified a lower birth weight, GA, and foetal growth restriction (FGR) as major factors associated with PH in preterm infants [14]. FGR is the inability of the developing foetus to grow to their genetic potential. FGR is defined as a birth weight (BW) of <10th percentile for GA and sex [32]. Infants born with FGR have a disproportionately higher risk of developing BPD. The factors which affect somatic growth may also affect pulmonary growth. Pulmonary artery thickness/stiffness has been noted in FGR in the early postnatal weeks and also in well-grown infants with established BPD. The lack of waveform cushioning by the major arteries exposes the smaller pulmonary arterioles to a higher pulsatile stress, thereby increasing the severity of microvascular disease [33].

A prospective echocardiographic study on Day 7 of preterm infants identified that early pulmonary vascular disease (PVD) was associated with the development of BPD and late PH. PVD was defined as an echocardiographic sign of PH if either one of the following criteria was present (an estimated right ventricular systolic pressure greater than 40 mmHg, any cardiac shunt with bidirectional or right-to-left flow, or a moderate to severe degree of ventricular septal wall flattening) [34]. The combination of PVD and the need for mechanical ventilation at Day 7 were strong predictors of respiratory problems in childhood [35]. Arjaans and colleagues have further characterised this early PH into three phenotypes based on shunting at the ductal level [36]. They reported that early PH in each of these phenotypes was associated with severe BPD. Persistent PH of the newborn (PPHN) phenotype was associated with early death. Whether the therapy targeted according to a particular phenotype helps in preventing injury to the pulmonary vasculature and mitigating the risk of BPD still remains to be answered.

High pulmonary blood flow is a major cause of haemodynamic stress on the pulmonary vasculature leading to PH in congenital heart disease. Similarly, a prolonged left-to-right shunt via a patent ductus arteriosus (PDA) and/or a significant atrial septal defect (ASD) may be important factors in preterm infants in the development of severe BPD and late PH. A recent Bayesian meta-analysis demonstrated that prolonged exposure to a PDA was associated with an increased risk of PVD in extremely preterm infants, suggesting the need to incorporate the risk of PH into the clinical decision regarding closure of the PDA [37]. The presence of a significant ASD was more commonly associated with PH in preterm infants with BPD as compared to those with BPD without an ASD [38,39]. Several recent studies have also highlighted the importance of chronic hypoxia contributing to the progression of PH. Jensen and colleagues in a post hoc analysis of the Canadian Oxygen Trial demonstrated that the relative risk of severe BPD rose with increased exposure and the duration of hypoxemic episodes [40]. Gentle et al. compared infants with BPD-PH with BPD alone and found that infants who developed PH had more prolonged hypoxemic exposures and those with readings of <70% saturation were associated with increased mortality [41].

Previous studies of BPD infants have focused on the pulmonary circulation—using right ventricular hypertrophy and the Doppler velocity of tricuspid regurgitation as markers of PH [11,15,16]. Data on the systemic (left sided) pathology were limited to the management of sHTN. However, a recent study highlighted the relationship of left-sided cardiac and vascular changes with the pathogenesis, diagnosis, and treatment of a subset of infants with BPD [42]. A higher incidence of sHTN was observed in infants with BPD [6,43,44,45]. Aortic stiffness has recently emerged as a possible pathophysiologic factor that may further contribute to an elevated afterload [42]. The pathophysiology of postcapillary PH may arise from chronic systemic arterial stiffness which in turn may generate sufficient afterload to induce left ventricle (LV) hypertrophy and/or dysfunction, high end-diastolic left atrial (LA) pressure, and subsequent pulmonary venous hypertension possibly contributing to pulmonary oedema [42,46]. In a recent study, our group has found diastolic dysfunction in a preterm BPD cohort characterized by elevated LA pressure accompanied by reduced pulmonary venous flow, thereby supporting the suggestion that the postcapillary pathology may be a contributor to the BPD pathophysiology [47].

### 3.3. Diagnosis of Bronchopulmonary Dysplasia Associated Pulmonary Hypertension (BPD-PH)

Multiple entities worldwide such as the American Heart Association and American Thoracic Society [48], the PPHNet [49], and the European Pediatric Pulmonary Vascular Disease Network (EPPVDN) [50] have provided consensus-based guidelines for the diagnosis, monitoring, and care of BPD-PH patients while acknowledging that there is a paucity of robust studies based on randomised clinical trials in this vulnerable infant population. The gold standard for diagnosing PH has been by cardiac catheterization [49]. However, it is rarely used because of its invasiveness, the difficulty of performing it in tiny patients, and its limited availability. Instead, non-invasive Echo has become the investigation of choice [51,52], further supported by a study utilising Echo which was able to correctly diagnose PH in children under the age of two years in 79% of cases [53].

**Figure 1:**
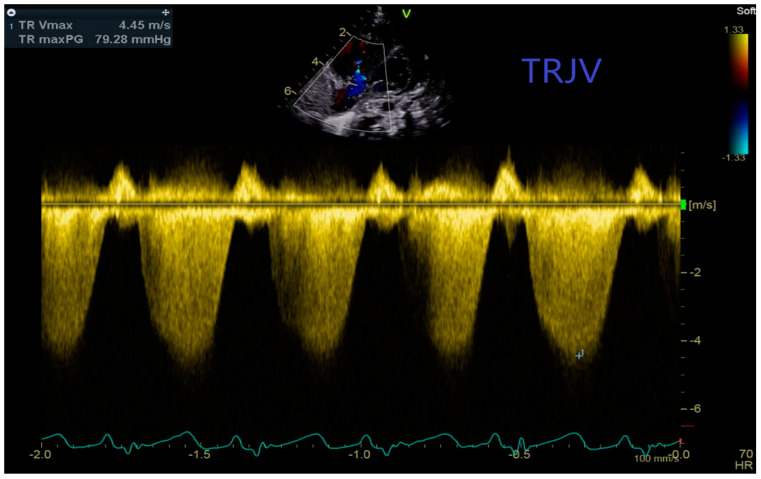
Continuous Doppler trace showing tricuspid regurgitant jet velocity (TRJV) Systolic pulmonary artery pressure estimated using the Bernoulli equation = 84 mmHg [PA pressure = 4 × (TRJV)^2^ + right atrial pressure (5 mmHg)].

Echo in addition to detailed imaging of the heart and the large vessels utilises Doppler-based methodology to estimate pressures [11]. Measurement of the tricuspid regurgitant jet velocity (TRJV) has been used to estimate the systolic pulmonary artery (PA) pressure using the Bernoulli equation [Peak PA pressure = 4 × (TRJV)^2^ + right atrial (RA) pressure]. A TRJV of 2.8 m/s plus an estimated RA pressure of 5 mmHg results in a peak PA pressure of 36 mmHg, i.e., the upper limit of normal [54,55]. Figure 1 shows a continuous Doppler trace of a TRJV of 4.45 m/s with an estimated PA pressure of 84 mmHg.

**Figure 2:**
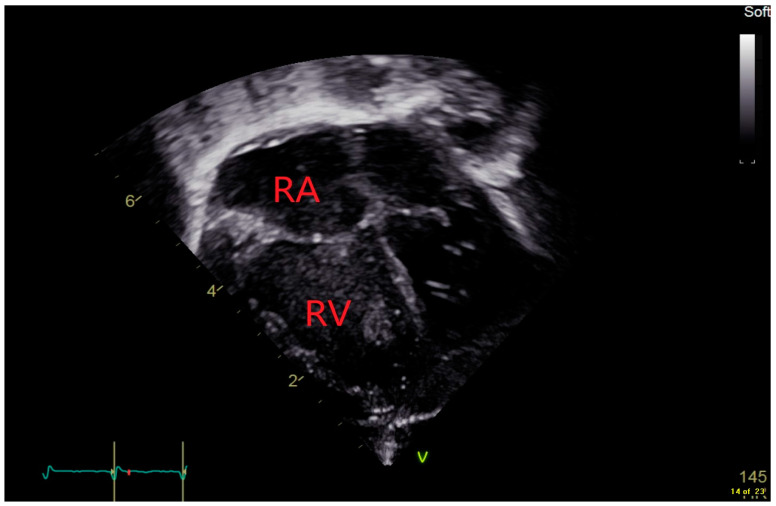
Apical 4-chamber view showing the dilated right atrium (RA) and right ventricle (RV).

TRJV is only measurable in 31–61% of infants with BPD with suspected PH because of the absence of a well-defined TRJV of tricuspid valve incompetence, while an absence of a measurable TRJV does not necessarily rule out PH [51,53]. To complement TRJV, additional Echo measurements such as RA enlargement, right ventricle (RV) hypertrophy and/or dilatation, septal flattening during systole, left ventricular systolic eccentricity index (LV sEI), and the time to peak velocity/right ventricular ejection time ratio (TPV/RVET) provide further markers of PH [51,52,56]. Figure 2 shows an apical four chamber view with a dilated RA and RV.

**Figure 3:**
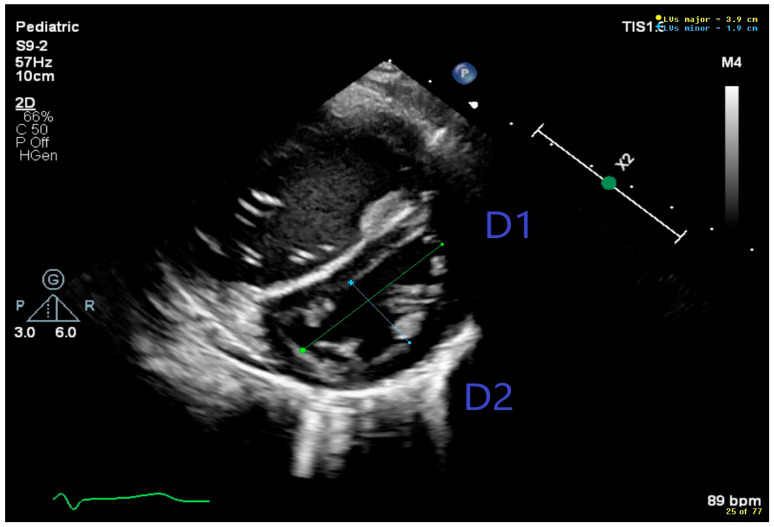
Short axis view of left ventricle showing flattening of the interventricular septum and high systolic eccentricity index (D1/D2) Eccentricity index in systole = 2 [D1(3.9 cm)/D2(1.9 cm) = 2, normal 1.2].

Figure 3 shows a parasternal short axis view of the LV with a flattened interventricular septum and a high LV sEI of 2; the normal eccentricity index is ≤1.2 [57].

**Figure 4:**
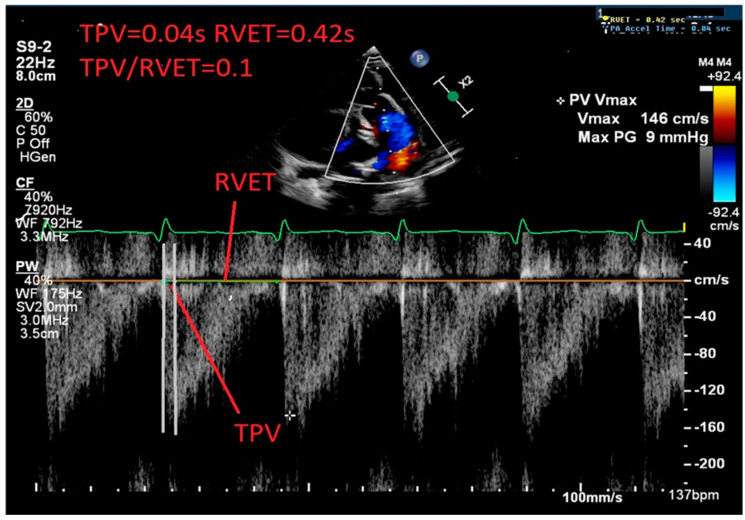
Pulmonary artery pulse Doppler trace showing a shortened time to peak velocity (TPV)/right ventricular ejection time ratio (RVET) TPV/RVET = 0.1 [TPV(0.04 s)/RVET(0.42 s) = 0.1, normal ≥ 0.31].

Figure 4 shows a PA Doppler trace with a significantly short TPV/RVET ratio of 0.1; the normal ratio is ≥0.31 [58].

There is a growing appreciation of the contribution of acquired pulmonary vein stenosis (PVS) in late PH. Its assessment should be part of every screening Echo for PH [59]. The EPPVDN 2019 consensus statement recommended a quantitative assessment of the RV by means of an age-related tricuspid annular plane systolic excursion (TAPSE) as a surrogate of longitudinal systolic RV function [50]. Non-invasive modalities of PH diagnosis including magnetic resonance imaging (MRI) and computed tomography (CT) have largely replaced cardiac catheterisation [50].

Serum brain-type natriuretic peptide (BNP) and its prohormone N-terminal cleavage product (NT-pro-BNP) levels are released by the myocardium in response to stretch. Changes in BNP or NT-pro-BNP levels are useful in monitoring the disease course and the response to clinical therapies over time [60,61,62]. However, these non-specific markers should be evaluated in conjunction with Echo [49].

## 4. When to Screen for PH?

Screening Echo for PH is recommended in any infant with established BPD at 36 weeks PMA, or those with prolonged oxygen requirement, poor growth, and an unsatisfactory clinical course. Follow-up studies for persistent PH should be performed every 2 weeks initially, to monitor the response to therapy, and then monthly [49,50].

### 4.1. Treatment of BPD-PH

The management of BPD-associated PH includes identifying and then treating the three major contributors to its development, namely the lung, the heart, and the pulmonary circulation. A multidisciplinary team approach is advisable involving the neonatologist, general paediatrician, respiratory physician, cardiologist, and allied health clinicians (dietician, occupational, and physiotherapist).

The PPHNet recommends treating the underlying lung disease, including carrying out an extensive evaluation for structural airway abnormalities such as tonsillar and adenoidal hypertrophy, vocal cord paralysis, subglottic stenosis, and tracheomalacia. Flexible bronchoscopy may be required to assess anatomic and dynamic airway abnormalities such as tracheomalacia, which may contribute to hypoxemia and poor clinical responses to oxygen therapy. Gastro-oesophageal reflux (GER) and micro-aspiration contribute to ongoing lung injury. Upper gastrointestinal investigations including pH or impedance probe monitoring, and swallow studies may be needed to evaluate GER [49].

### 4.2. Oxygen Therapy

The current recommendation for the treatment of BPD-PH suggests an avoidance of oxygen saturations below 92%, aiming to maintain levels of 94% to 96%. Supplemental oxygen should be administered to maintain target oxygen saturations of >93% for infants with suspected or proven PH [63,64].

### 4.3. Nutrition

Growth failure is a common problem in infants with established BPD. Nutrition should aim to provide adequate calories and nutrients to establish continued growth and assist with lung alveolarization. Infants with severe BPD have a higher caloric requirement. The European Society of Pediatric Gastroenterology, Hepatology, and Nutrition (ESPGHAN) Committee on Nutrition 2022 recommends a total energy intake of 115–140 kcal/kg/d for most growing preterm infants to achieve optimal growth [65]. It also states that energy intakes > 140 kcal/kg/d may be necessary where growth is below the recommended range but should not be provided until protein and other nutrient sufficiency has been achieved. The intake should not exceed 160 kcal/kg/d. However, there are no specific recommendations on energy requirements for infants with severe BPD. It is imperative that dieticians are consulted to optimise the nutritional intake of affected babies.

Miller and colleagues have classified established BPD into three phases, each with differing nutritional needs [66]. The acute phase I (high oxygen need, steroid, PH) requires a higher caloric (120–150 kcal/kg/d) and protein intake (4 g/kg/d), followed by a transitional phase II (weaning oxygen, steroid, and a falling PH) requiring a reduced caloric (110–120 kcal/kg/d) and protein intake (2.5 to 4 g/kg/d). The pro-growth phase III (stable oxygen, off steroid) where BPD and BPD-PH are improving requires a reduced caloric (70–120 kcal/kg/d) and protein intake (2.5–3 g/kg/d). A significant improvement in all growth parameters was demonstrated using this strategy.

### 4.4. Diuretics

The European consensus PH guidelines have recommended diuretic therapy (hydrochlorothiazide and spironolactone) as a first-line treatment for BPD-PH [50]. Nonetheless, diuretic therapy is not widely used due to its negative effect on growth and metabolic bone disease. However, the diuretic therapy was associated with improvement of the pulmonary vascular resistance, right and left ventricular function and compliance, although there was transient hyponatremia in >60% infants and a decreased growth velocity [67].

### 4.5. Pharmacotherapy

The aim of the pharmacotherapy is to increase pulmonary blood flow by achieving a reduction in pulmonary vascular resistance. It targets three main pathways: nitric oxide, the endothelin receptor, and prostacyclin. The commonly used pulmonary vasodilators include inhaled nitric oxide (iNO), sildenafil, bosentan, and prostacyclin. Their use in infants is off-label. The Food and Drug Administration (FDA) has approved bosentan to treat PH in children >3 years old. The European Medicines Agency (EMA) has approved both sildenafil (>1 year old and 8 kg weight) and bosentan (>1 year old) for the treatment of PH [63].

**iNO** therapy for PH was pioneered by Abman and Kinsella [68]. NO is a potent vasodilator, which induces cyclic guanosine monophosphate (cGMP) to promote vascular smooth muscle relaxation [69]. However, recent trials have shown that iNO has limited or no efficacy for preventing BPD [70]. iNO is not approved by FDA for BPD-PH. PPHNet guidelines recommend iNO to be used as a rescue therapy for acute PH crises for infants with BPD-PH, to be weaned once stabilisation is achieved [49].

**Sildenafil** is a selective phosphodiesterase type 5 (PDE5) inhibitor that induces NO-mediated vascular relaxation and suppresses smooth muscle proliferation [71]. Sildenafil also preserves lung angiogenesis and decreases PVR and media wall thickness [72]. It reduces the pulmonary inflammatory response and fibrin deposition, making it an ideal drug for BPD infants with PH [73]. Despite a lack of strong evidence of its efficacy and regulatory approval, sildenafil is routinely used off-label to treat BPD-PH [74]. In a retrospective study involving sildenafil administration to 22 infants with a diagnosis of BPD-PH, our group reported a significant improvement in the echocardiographic indices of PH [75]. A recent systematic review and meta-analysis evaluated five studies (n = 101) of infants with a mean GA of 26 weeks with BPD-PH for the effectiveness of the long-term use of sildenafil. The PA pressure improved by >20% in the majority of cases within 1–6 months. It did not affect mortality and there were no serious adverse events [76].

**Bosentan** is a non-selective antagonist of the endothelin-1 (ET-1) receptors ET-A and ET-B. It has potent vasodilatory effects, thereby reducing pulmonary vascular resistance [77]. Bosentan is widely and effectively used to treat PH in adult patients. Bosentan use in paediatric patients with pulmonary arterial hypertension has resulted in hemodynamic improvement; however, elevated liver aminotransferases may occur and represent a serious adverse complication [78,79,80]. Therefore, monthly liver function studies are required with its long-term use, with additional testing in the event of an intercurrent viral infection. There is limited evidence of the efficacy of bosentan use for BPD-PH with no long-term studies to guide the therapy [52,81].

**Prostacyclin (PGI_2_)** is endogenously produced by the vascular endothelium. PGI_2_ binds to its receptor, stimulating adenylate cyclase to produce intracellular cyclic adenosine monophosphate (cAMP), resulting in smooth muscle relaxation [82]. Epoprostenol, a synthetic version of PGI_2_, was one of the earliest prostacyclins used for PH. Its short half-life means it needs continuous infusion and requires central line access. In adults and children with idiopathic pulmonary hypertension, epoprostenol has been shown to improve the pulmonary haemodynamics, quality of life, exercise capacity, and survival of those affected [83,84]. The efficacy and safety of this therapy is still limited in infants with BPD-PH [85,86]. Its use is limited to hospitalised infants, as it requires a continuous infusion with dedicated intravenous access.

A summary of the pulmonary vasodilator medications and their recommended dosage is provided in Table 1 [49,50].

## 5. Promising New Therapies for BPD-PH

**Melatonin** is a lipid soluble neuroprotective antioxidant. It has vasodilator properties which may be effective at a pulmonary level [87]. The daily administration of melatonin in a chronically hypoxic rat model significantly lowered the RV systolic pressures, thickness of the arteriolar wall, and oxidative and inflammatory markers [88]. A further study with its postnatal use in lambs with chronic hypoxia reduced the pulmonary oxidative stress by inducing antioxidant enzymes and improved the pulmonary vascular reactivity [89]. These positive responses need further exploration in human studies.

**Interleukin-1 receptor antagonist (IL-1Ra)** is another therapy under consideration. Interleukin-1 (IL-1), a potent inflammatory cytokine, is recognised as a key factor in the development of BPD and BPD-PH. IL-1Ra is a natural protein that blocks IL-1 and has been safely used as a drug (Anakinra) to treat diseases such as rheumatoid arthritis, gout, and heart failure [90]. Animal studies have shown that blocking inflammation early with Anakinra may help by preventing BPD and BPD-PH and improving neurodevelopmental outcomes [91,92,93]. Anakinra is currently undergoing a safety and feasibility phase I/IIa dose escalation trial in extremely preterm infants.

**L-Citrulline** is an amino acid precursor of the nitric oxide substrate L-arginine. It increases the intracellular production of L-arginine which in turn increases the amount of NO produced by the pulmonary vascular endothelial cells [94]. Citrulline has shown promising results in a newborn piglet model in preventing the development of hypoxia induced PH by increasing nitric oxide production [95]. Recently, a pharmacokinetic study of an enterally administered single dose L-citrulline in 10 preterm neonates at risk of developing BPD-PH showed a good tolerance [96]. These findings have led to a phase II study to evaluate its safety and potential efficacy.

### 5.1. Long-Term Respiratory Outcomes of BPD-PH

A few studies have reported respiratory and feeding problems associated with BPD-PH. Mourani and colleagues evaluated the association of BPD-PH and respiratory morbidities during childhood. Infants mechanically ventilated and shown to have PVD at 7 days had 8-fold increased odds of having BPD exacerbation, asthma, bronchiolitis, pneumonia, or a respiratory-related hospitalization during a 2-year follow-up. The diagnosis of PH and BPD at 36 weeks was not as useful as PH at Day 7 in predicting late respiratory morbidities [81]. Altit and colleagues reported significant feeding problems in their cohort of 61 infants with BPD-PH. Aspiration was found in 38% patients on a videofluoroscopic swallowing study, and 44% of the cohort needed a gastrostomy. Most of the patients (75%) had rehospitalisation after discharge and 71% of those required at least one paediatric intensive care (PICU) admission. Chest CT showed abnormal lung parenchyma in most of the cohort [97]. Lagatta and colleagues in their large multicentre cohort study of patients with severe BPD reported that PH was associated with an increased need for a tracheostomy, home oxygen, tube feeding, and an increased frequency of readmission in their first year [98].

### 5.2. Long-Term Neurodevelopmental Outcomes of BPD-PH

Preterm infants with BPD are at a high risk for poor neurodevelopmental (ND) outcomes throughout childhood [99]. However, there is limited information on the ND outcomes of infants with BPD-PH. A recent study by Thomas and colleagues examined the ND outcomes using the Bayley Scales of Infant and Toddler Development, Third Edition (BSID-III), of preterm infants born at <29 weeks GA with BPD-PH at 18 to 24 months PMA. BPD-PH infants had a 3.8-fold increased odds of having a composite outcome of death or ND impairment (Bayley-III score <85 on one or more of the cognitive, motor, or language assessments) [100]. Similarly, prospectively collected data from the Korean Neonatal Network suggested that BPD-PH is an independent risk factor associated with an increased risk of mortality or ND delay (adjusted OR 1.95, CI 1.17–3.25) [101]. Nakanishi and colleagues noted BPD-PH infants were more likely to have a developmental quotient (DQ) in the significantly delayed range (DQ < 70) compared with BPD alone at an ND assessment at 3 years of age [102]. Choi and colleagues found that compared to BPD infants, BPD-PH infants showed significantly lower cognitive, language, and motor scores on the BSID-III at 18–24 months PMA. Cognitive delay was found in 45% of PH infants [103]. These findings suggest the importance of long-term follow-ups to facilitate the early recognition of developmental issues and the need for timely interventions to optimize the outcomes of affected infants.

### 5.3. Survival of BPD-PH Infants

The reported survival rate in BPD-PH infants ranges from 53 to 72% [16,97,98,104,105]. Khemani and colleagues in their cohort of 42 preterm infants with BPD-PH reported survival rates of 64% at 6 months and 53% at 2 years. The severity of PH and FGR were associated with poor survival. Among survivors, their PH improved in most of them (90%) at a median follow-up of 10 months [16]. Similarly, Arjaans and colleagues reported a low survival rate of 58% at 6 months PMA. Suprasystemic PH was associated with a lower survival. However, there was no additional mortality after the initial period during a median 2.8-year follow-up, suggesting a favourable outlook regarding survival beyond 6 months of age. In surviving infants, PH resolved over 2.5 years [105]. Altit et al. studied PH in infants ≤ 32 weeks GA and identified the male gender, the severity of the PH, and the use of postnatal steroids as risk factors for death. Amongst their cohort at a mean age of 5 years, 72% survived. Two-thirds of infants had resolution of their PH at a median age of 3 years [97].

### 5.4. Long-Term Cardiovascular Outcomes of BPD-PH

PH resolution does not suggest complete normalisation of the pulmonary vasculature. Infants born preterm with early PVD are still at risk of later cardiac and respiratory morbidities in childhood and as young adults where there is growing evidence of PVD. PA pressures were estimated to be greater in 11- to 14-year-old children born extremely premature compared with those born at term [106]. Mildly elevated PA pressures, a stiffer pulmonary vascular bed, RV dysfunction [107], and impaired ventriculo-pulmonary vascular coupling [108] contribute to the risk of developing PH in young adults if born prematurely. Cardiac MRIs of such adults have shown smaller LV chamber sizes with thickened ventricular walls, an elevated systemic vascular resistance, and a diastolic functional impairment at a younger age [109]. These findings suggest the need for close long-term cardiovascular and pulmonary function monitoring for BPD-PH infants. Early risk factors need to be identified to avoid late cardio-respiratory impairment through appropriate preventive treatment strategies to improve outcomes. Box 1 summarises the cardiovascular and neurodevelopmental outcomes of infants with BPD-PH.

Box 1 **Short and long-term outcomes of BPD-PH infants.**

- BPD-PH infants have a high mortality rate (28–47%) during their initial hospitalisation and early infancy.
- It is associated with significant feeding problems such as aspiration, GER, nasogastric, and gastrostomy feeding.
- Respiratory problems include the need for home oxygen, tracheostomy, and frequent admissions during the first year of life due to asthma, bronchiolitis, pneumonia, and exacerbations of BPD.
- They are at risk of poorer ND outcomes resulting in lower cognitive, language, and motor scores. Most have a significantly delayed developmental quotient.
- Most infants show resolution of their PH with ongoing therapy; however, the cardio-respiratory sequelae persist in young adults who require long-term follow-up.

## 6. BPD-Ssociated Systemic Hypertension (BPD-sHTN)

Systemic hypertension (sHTN) in neonates was first described in the late 1970s with its awareness increasing over time [110]. Newborns’ BP increases with GA and birth weight [111]. Birth weight and PMA are the most important predictors of BP in early life [112]. Neonatal sHTN is defined as a systolic and/or diastolic BP > 95th percentile based on Dionne and colleagues’ published data for systolic and diastolic BP percentiles for 26 to 44 weeks PMA [113]. The prevalence of sHTN varies from 0.2 to 3% in the neonatal intensive care unit (NICU) [114]. The cause of sHTN varies widely and is related to unique perinatal risk factors such as renal artery thrombosis [115] (secondary to thromboembolism from umbilical catheters) [116], medication—administration of steroids [117], and indomethacin and acute renal failure [116]. However, the prevalence of idiopathic hypertension (5–57%), where no cause can be found, is still significant [116,118,119].

sHTN seems to be a significant factor in BPD infants, with important implications for its management and prognosis. Preterm infants with BPD are at a higher risk of developing sHTN compared to other preterm neonates [120]. BPD-sHTN was first described in the mid-1980s by Abman, who reported 43% sHTN rates in infants with BPD compared to an incidence of 4.5% in infants without BPD. Over half of the infants with BPD and HTN were not diagnosed until after discharge from the NICU, emphasizing the importance of ongoing monitoring of their BP [6].

### 6.1. Pathophysiology of BPD-sHTN

The association between BPD and sHTN has been known for several decades; however, it remains unclear whether there is a mechanistic pathway by which BPD directly leads to the development of sHTN, or whether BPD is simply a surrogate risk factor for other conditions, including sHTN.

sHTN complicates a subset of infants with BPD and has been associated with a longer duration of respiratory support and hospital stay as well as a higher mortality [44]. Factors contributory to BPD pathophysiology such as pro-inflammatory cytokines, oxidative stress, and higher catecholamine levels can lead to systemic arterial remodelling. Inflammation and oxygen toxicity are known to adversely affect vascular function through abnormal collagen deposition and endothelial dysfunction [121]. In addition, sympathetic over-activity could be a key link for sHTN and may result from a reduced clearance of catecholamines by the pulmonary circulation [122]. A recent study found the abdominal aorta was thicker with a higher impedance, stiffness, and vascular resistance amongst the BPD infants [42]. The combination of increased catecholamines and systemic arterial stiffness may act synergistically to generate a sufficient afterload which contributes to the development of sHTN. Aortic arterial stiffness may lead to an increased afterload, decreased myocardial relaxation, LV diastolic dysfunction, and elevated end diastolic LA pressure. These physiologic changes may contribute to pulmonary venous congestion and oedema, leading to reduced lung compliance. The clinical consequences include the prolonged need for respiratory support, inflicting further baro/volutrauma on the developing lung [42].

Arterial stiffness may have important distal effects as well. The aorta is the conduit between the LV and the peripheral vascular bed, it dampens the intermittent pressure waveform generated by the LV. This results in the delivery of a continuous and steady blood flow distally. Reduced cushioning exposes the distal vasculature to a higher pulsatile stress, thereby contributing to end-organ microvascular renal disease [123]. sHTN results in attenuation of the microvascular arteriolar network, disturbing the cardiac–vasculature coupling and potentiating cardiac dysfunction [124]. FGR, a well-known risk factor for BPD, may contribute to sHTN via an accelerated vascular aging and an activated renin–angiotensin system (RAS) leading to a rise in vascular resistance [125].

Abnormal cardiovascular development continues in young adults. Flahault and colleagues reported that young adults born preterm with BPD display alterations in arterial distensibility and a reduced ascending aorta diameter, which contribute to an increased diastolic blood pressure [126]. Cardiac MRIs in adults born preterm show abnormalities of LV mass, geometry, and function [127]. These findings may be related to a decreased cardiomyocyte proliferation and maturation [128]. Myocyte differentiation occurs in the third trimester and the heart loses its capacity to proliferate soon after birth. Therefore, the preterm immature myocardium is at risk of maldevelopment. Recent adult studies have shown that individuals born preterm are at a higher risk of developing sHTN and LV maldevelopment including myocardial fibrosis [127,129].

### 6.2. Prevalence of BPD-sHTN

There are limited data regarding the prevalence of sHTN in association with BPD [6,43,44]. Abman and colleagues diagnosed sHTN if the systolic BP was >113 mmHg [6]. In this retrospective study, 13/30 (43%) infants with BPD demonstrated sHTN. The mean age of onset ranged from 0.5 to 15 months, and more than half were diagnosed after NICU discharge. Anderson et al., in a retrospective study of 87 infants with BPD, diagnosed sHTN in 11/87 (13%) infants with the mean age of onset being 6 months [44]. A recent retrospective study defined sHTN when three separate measurements of systolic BP were >95th centile, and over a 4-year period, 53 (1.3%) infants had hypertension; of whom 74% were preterm. BPD was identified as a major risk factor [130].

Our group, in a retrospective study, assessed the correlation between sHTN and respiratory complications amongst infants with BPD utilising the Dionne BP charts. A six-year dataset compared 57 infants with severe BPD to 114 infants with no BPD. In total, 40% infants (23/57) had sHTN in the BPD cohort compared to 2.6% of controls (3/114). Amongst the BPD infants, sHTN was associated with a longer duration of respiratory support. There was no assessment of RAS [45]. Recently, Reyes-Hernandez et al. reported the incidence of sHTN in 47% (30/64) of their extremely preterm infants (<28 weeks) with BPD and PVD [131]. sHTN was defined as a systolic BP ≥ 90 mmHg at the time of the Echo. Hypertensive infants showed LV diastolic dysfunction and left heart volume and pressure loading. They also had a higher pulmonary vascular resistance index. These findings suggest a potential link between sHTN, LV diastolic dysfunction, and the risk of PVD leading to postcapillary pulmonary congestion and prolonged respiratory support. sHTN infants had a higher diuretic use but only two infants were treated with antihypertensive therapy (amlodipine). The renal function or RAS of their cohort were not documented.

Similarly, in a larger multicentre study of preterm infants (<37 weeks) with sHTN, Jenkins et al. reported that 38% of their cohort had BPD [119]. Of those with BPD and sHTN, infants were diagnosed with sHTN at an average age of 40 weeks PMA and had resolution of their sHTN by 1 year of age, and 98% had low plasma renin activity (PRA). Spironolactone was the most commonly used diuretic therapy as an antihypertensive treatment. This long-term study over 16 years did not find a recurrence of sHTN in this cohort.

All of the above studies demonstrated a wide range in the frequency of sHTN. There was a heterogeneity in the definition of BPD, and different cutoffs and timelines were used for BP recordings. The American Academy of Pediatrics 2017 Clinical Practice Guideline Subcommittee on the Screening and Management of High Blood Pressure in Children recommended the use of derived BP centiles based on PMA [132].

### 6.3. Diagnostic Approach for Assessment of BPD-sHTN

Accurate and reliable BP measurement is essential to correctly identify sHTN in neonates. The gold standard for the measurement of BP in neonates is arterial catheter measurement [113]. However, in clinical practice, indirect methods using oscillometric devices are more common and practical. There is a good correlation between invasive monitoring and oscillometric assessment; however, the latter tends to overestimate BP [133]. In infants with BPD-sHTN, a detailed history of antenatal factors, medications, kidney injury, and interventions (umbilical arterial catheter) is needed to identify alternate causes. A detailed clinical examination is required to identify any organ-specific pathology. Careful attention should be given to palpate peripheral pulses (especially the femorals) and differential BP to identify arch abnormalities [134]. If there is a clinical concern for coarctation, an Echo assessment of the aortic arch is recommended [135]. Laboratory testing should be performed to review renal function and to check the urine for protein, creatinine, and microalbumin in order to ascertain possible renal parenchymal disease. Other tests such as the determination of cortisol, aldosterone, and thyroxine levels may be required if clinically indicated. Ultrasound imaging with Doppler of the genitourinary tract should be obtained in all HTN infants [113].

### 6.4. Therapeutic Options for BPD-sHTN

In BPD-sHTN underlying arterial stiffness, increased activation of the RAS and the vasoconstricting and fibrotic actions of angiotensin II on the vasculature may have an important role to play. Inhibition of the RAS system by angiotensin converting enzyme (ACE) inhibitors may be an important therapeutic option. ACE inhibitors act by resetting the balance between vasoconstriction/proliferation (angiotensin II, reactive oxygen species, endothelin-I) and vasodilatation/antiproliferation (bradykinin, NO) [136,137,138,139,140,141]. In a recent study of adult patients, although all the agents were equally effective in reducing BP, only ACE inhibitors improved endothelial function compared with calcium antagonists, beta blockers, and diuretics [142]. The blockade of the RAS system has a significant impact on arterial structure and function independent of BP [143]. The inhibition of arterial smooth muscle hypertrophy and the inhibition of elastin fibre replacement by collagen fibres in large arteries may mediate this response [144]. Collagen is 100 times stiffer than elastin which results in a reduced arterial compliance and an increased afterload [145]. Thus, ACE inhibitors exhibit a vascular remodelling effect which is evident by the concurrent reduction in the systemic afterload, enhanced LV function (especially diastolic function), and a reduction in LV end diastolic pressure, thereby improving the pulmonary venous return. We routinely use an ACE inhibitor (captopril) as the first-line therapy for neonatal sHTN [45]. Therapy is commenced after 37 weeks PMA. Electrolytes and renal function are monitored weekly.

An ACE inhibitor also benefits a select population of BPD-sHTN associated with LV diastolic dysfunction. Mourani and colleagues [46] described two infants with PH and LV dysfunction unresponsive to diuretics and iNO therapy. Captopril therapy led to clinical improvement, the normalization of Echo parameters, and subsequent discharge home. A recent study demonstrated clinical and echocardiography improvement, following the initiation of captopril in six infants with severe BPD-sHTN unresponsive to steroids, diuretics, and sildenafil [146]. Systolic BP (>100 mmHg in all cases) normalised after 5 weeks along with a reduction in oxygen (55% to 29%) and ventilator requirements. This response coincided with improved LV function, a reduction in aortic intimal media thickness, and an increased aortic pulsatile diameter. Similarly, a small cohort of infants (n = 11) with PVD secondary to BPD-sHTN manifesting LV diastolic dysfunction but maintained systolic function showed improvement from the ACE inhibitor Enalapril [147]. These findings support the idea that the modification of the systemic vascular haemodynamics may reduce PH and improve cardiovascular health in infancy and early childhood.

Other antihypertensive agents may be used to treat sHTN and include calcium channel blockers, alpha and beta-blockers, central alpha agonist, diuretics, and direct vasodilators and are summarised in Table 2 [113,120].

### 6.5. Long-Term Outcomes of BPD-sHTN

There is a paucity of studies regarding the long-term course of sHTN. The available data are reassuring in that BPD-associated sHTN tends to resolve over a few years, with most subjects off medication after a year [6,119]. Altit et al. evaluated the ND outcomes of infants with BPD-sHTN in a retrospective study of preterm infants (<29 weeks). The ND outcomes (BSID-III) at 18 months were similar in infants with sHTN compared to preterm infants without sHTN. After adjusting for confounders in a regression model, sHTN was not associated with ND impairment [148]. Box 2 summarises the cardiovascular and neurodevelopmental outcomes of infants with BPD-sHTN.

Box 2 **Short and long-term outcomes of BPD-sHTN infants.**

- Approximately 40% of BPD infants develop BPD-sHTN.
- Standardised BP percentile charts are necessary for diagnosis and appropriate management.
- Infants with BPD-sHTN generally require longer respiratory support.
- ACE inhibitors and diuretics seem to improve cardiovascular indices.
- Medication therapy is required during the first year.
- ND outcomes may be similar to BPD infants without sHTN.

## 7. Conclusions

BPD and its devastating complications BPD-PH and BPD-sHTN continue to be the major burden of extreme prematurity. Both disorders are driven by chronic pulmonary inflammation. Multiple causative links between hypoxia, FGR, BPD, PH, and sHTN have been provided. Although pulmonary vasodilators are the mainstay of PH therapy, afterload reducing agents (ACE inhibitor) may be helpful for those with sHTN especially if there is evidence of postcapillary PH. Preterm infants with BPD-PH carry a higher mortality, particularly within their first year. They are also at risk of long-term cardio-respiratory and gastrointestinal problems and require a multidisciplinary approach. A detailed echocardiography of infants with severe BPD, BPD-PH, and BPD-sHTN ensures an important baseline assessment and aids in long-term monitoring.

## 8. Directions for Future Research

Further longitudinal multicentre studies are required to investigate the short- and long-term outcomes of BPD-PH and BPD-sHTN. All infants with severe BPD require a thorough assessment of both right and left heart, systolic, and diastolic function at 36 weeks PMA to further understand their pathophysiology. Further research needs to focus on identifying anti-inflammatory treatments that may target BPD at an early stage to alter its natural history and its long-term complications. Similarly, longitudinal studies are required to understand the natural history of BPD-sHTN and its impact on renal and cardiovascular outcomes into adulthood. A better understanding of the pathophysiology of both BPD-PH and BPD-sHTN may allow for more targeted and effective therapy.

## Figures and Tables

**Table 1:** Pulmonary vasodilators used for broncho-pulmonary dysplasia-associated pulmonary hypertension (BPD-PH).

Name	Dose	Side Effects
iNO (cGMP augmentation)	Initial dose of 10–20 PPM for acute PH crises and wean after stabilization to a dose of 3–5 PPM followed by a gradual reduction by 1 PPM before ceasing	Rebound PH after abrupt cessation. Can be minimised with gradual weaning and adding PDE5 inhibitor
Sildenafil (PDE-5 inhibitor)	Oral: 1 mg/kg 6–8 h; commence with a low dose (0.3–0.5 mg/kg/dose) and increase gradually to 1 mg/kg/dose as tolerated; Maximal dose of 10 mg q 8 h per EMA guidelines for infants	Hypotension, GER, irritability, bronchospasm, nasal stuffiness, fever
Bosentan (ET-1 antagonist)	Oral:1 mg/kg q 12 h as starting dose; may increase to 2 mg/kg q 12 h in 2–4 weeks, if tolerated and normal liver enzymes	Liver dysfunction especially during viral infections, hypotension, anaemia, oedema, and airway issues
Epoprostenol (cAMP augmentation)	Intravenous: commence at 1–2 ng/kg/min, titrate up slowly every 4–6 h to 20 ng/kg/min; Further increased if clinically indicated and no adverse effects	Hypotension, VQ mismatch, GI disturbances. Very short half-life with high risk for rebound PH with brief interruption of therapy Needs dedicated line

cAMP—cyclic adenosine monophosphate; cGMP—cyclic guanosine monophosphate; ET-1—endothelin-1; GER—gastroesophageal reflux; GI—gastrointestinal; iNO—inhaled nitric oxide; kg—kilogram; mcg—microgram; ng—nanogram; PDE5—phosphodiesterase Type 5; VQ—ventilation–perfusion. Adapted from Krishnan and colleagues for the Pediatric Pulmonary Hypertension Network [49].

**Table 2:** Antihypertensive medications used for the treatment of systemic hypertension (sHTN).

Name	Dose	Side Effects
Captopril (ACE inhibitor)	Oral: <3 m: 0.01–0.5 mg/kg/dose, TID Max 2 mg/kg/day >3 m: 0.15–3 mg/kg/dose Max 6 mg/kg/day	First-dose hypotension especially if receiving diuretics concomitantly Monitor electrolytes and renal function test
Enalapril (ACE inhibitor)	Oral: 0.08–0.6 mg/kg/day, OD or BID	Intravenous enalapril is not recommended as it may cause prolonged hypotension and oliguric acute renal failure. Monitor electrolytes and renal function test
Amlodipine (calcium channel blockers)	Oral: 0.05–0.3 mg/kg/dose, OD max 0.6 mg/kg/day	Mild reflex tachycardia
Propranolol (β-antagonists)	Oral: 0.5–1.0 mg/kg/dose, TID Max 8–10 mg/kg/day	Monitor heart rate, avoid use in BPD
Labetalol (α- and β-antagonists)	Oral: 0.5–1.0 mg/kg/dose, BID or TID Max 10 mg/kg/day	Heart failure
Clonidine (central α-agonist)	Oral: 5–10 mcg/kg/day, TID max 25 mcg/kg/day	May cause mild sedation
Hydrochlorothiazide (diuretic)	Oral: 1–3 mg/kg/dose, OD	Monitor electrolytes, beneficial in BPD
Spironolactone (diuretic)	Oral: 0.5–1.5 mg/kg/dose, BID	Monitor electrolytes, beneficial in BPD
Hydralazine (direct vasodilator)	Oral: 0.25–1.0 mg/kg/dose, TID or QID Max 7.5 mg/kg/day	Tachycardia and fluid retention

ACE—angiotensin converting enzyme; BID—twice daily; Max—maximum; QD—once daily; QID—four times daily; TID—three times daily. Reproduced with permission from Dionne and colleagues, Pediatr Nephrol; published by Springer Nature, 2012 [113].

## Abbreviations

ACE	Angiotensin Converting Enzyme
ASD	Atrial Septal Defect
ANZNN	Australian New Zealand Neonatal Network
BSID-III	Bayley Scales of Infant and Toddler Development, Third Edition
BW	Birth Weight
BP	Blood Pressure
BNP	Brain Natriuretic Peptide
BPD	Broncho-Pulmonary Dysplasia
CT	Computed Tomography
cAMP	Cyclic Adenosine Monophosphate
cGMP	Cyclic Guanosine Monophosphate
ET-1	Endothelin-1
EMA	European Medicines Agency
EPPVDN	European Paediatric Pulmonary Vascular Disease Network
FDA	Food and Drug Administration
FGR	Foetal Growth Restriction
GER	Gastro-Oesophageal Reflux
GA	Gestational Age
iNO	Inhaled Nitric Oxide
IL-1	Interleukin-1
IL-1Ra	Interleukin-1 Receptor Antagonist
LA	Left Atrium
LV	Left Ventricle
LV sEI	Left Ventricular Systolic Eccentricity Index
MRI	Magnetic Resonance Imaging
NT-pro-BNP	N-Terminal Pro Brain Natriuretic Peptide
NICU	Neonatal Intensive Care Unit
ND	Neurodevelopmental
NO	Nitric Oxide
PDA	Patent Ductus Arteriosus
PPHNet	Pediatric Pulmonary Hypertension Network
PDE5	Phosphodiesterase Type 5
PMA	Post-Menstrual Age
PGI2	Prostacyclin
PA	Pulmonary Artery
PH	Pulmonary Hypertension
PVS	Pulmonary Vein Stenosis
PVD	Pulmonary Vascular Disease
PVR	Pulmonary Vascular Resistance
RAS	Renin–Angiotensin System
RA	Right Atrium
RV	Right Ventricle
sHTN	Systemic Hypertension
TPV/RVET	Time To Peak Velocity/Right Ventricular Ejection Time Ratio
TAPSE	Tricuspid Annular Plane Systolic Excursion
TRJV-	Tricuspid Regurgitant Jet Velocity
VEGF	Vascular Endothelial Growth Factor
V/Q	Ventilation–Perfusion ratio
